# The influence of inhaled multi-walled carbon nanotubes on the autonomic nervous system

**DOI:** 10.1186/s12989-016-0119-7

**Published:** 2016-02-11

**Authors:** W. Zheng, W. McKinney, M. Kashon, R. Salmen, V. Castranova, H. Kan

**Affiliations:** 1 Health Effects Laboratory Division, Pathology and Physiology Research Branch, National Institute for Occupational Safety and Health, 1095 Willowdale Road, Morgantown, WV 26505 USA; 2 Department of Physiology and Pharmacology, West Virginia University, Morgantown, WV 26505 USA; 3 Department of Pharmaceutical Sciences, West Virginia University, Morgantown, WV 26505 USA

**Keywords:** Inhalation, Multi-walled carbon nanotubes, Autonomic nervous system, Heart rate variability

## Abstract

**Background:**

Heart rate and cardiovascular function are regulated by the autonomic nervous system. Heart rate variability (HRV) as a marker reflects the activity of autonomic nervous system. The prognostic significance of HRV in cardiovascular disease has been reported in clinical and epidemiological studies. The present study focused on the influence of inhaled multi-walled carbon nanotubes (MWCNTs) on autonomic nervous system by HRV analysis.

**Methods:**

Male Sprague–Dawley rats were pre-implanted with a telemetry device and kept in the individual cages for recovery. At week four after device implantation, rats were exposed to MWCNTs for 5 h at a concentration of 5 mg/m^3^. The real-time EKGs were recorded by a telemetry system at pre-exposure, during exposure, 1 day and 7 days post-exposure. HRV was measured by root mean square of successive differences (RMSSD); the standard deviation of inter-beat (RR) interval (SDNN); the percentage of successive RR interval differences greater than 5 ms (pNN5) and 10 ms (pNN10); low frequency (LF) and high frequency (HF).

**Results:**

Exposure to MWCNTs increased the percentage of differences between adjacent R-R intervals over 10 ms (pNN10) (*p* < 0.01), RMSSD (*p* < 0.01), LF (*p* < 0.05) and HF (*p* < 0.01).

**Conclusions:**

Inhalation of MWCNTs significantly alters the balance between sympathetic and parasympathetic nervous system. Whether such transient alterations in autonomic nervous performance would alter cardiovascular function and raise the risk of cardiovascular events in people with pre-existing cardiovascular conditions warrants further study.

## Background

With potential wide industrial applications, the impacts of airborne engineered nanomaterials on human health have recently raised significant concerns to investigators. The studies have found that due to their small size (nanometer range), unique electrical, mechanical, and thermal properties, inhalation of engineered nanoparticles is possible and may induce a series of adverse effects not only in the lungs, but other organs as well [1–3]. We and other investigators reported previously that inhalation of nanoparticles, such as ultrafine titanium dioxide (UFTiO_2_), can exhibit impacts on vascular constriction, blood pressure and cardiac function [4, 5]. There are three major accepted hypothetic mechanisms elucidating how inhaled nanoparticles could affect the cardiovascular system. These hypothetic mechanisms include a local inflammation-mediated reaction, the particle translocation, and a neuronal-regulated pathway [6–9]. In our previous study, our results indicated that the effects on the cardiovascular system induced by inhalation of UFTiO_2_ were most likely through an autonomic neuron-regulated pathway [4, 9]. This conclusion was based on our findings that inhalation of UFTiO_2_ can stimulate pulmonary C-fiber sensory neurons and enhance activity of neural transmitter synthesis in nodose ganglia [9]. Nerve fibers from the nodose ganglia project to the brainstem, including the medullar cardiovascular regulatory center, to regulate autonomic efferent neuron activity [10, 11]. Therefore, stimulation of peripheral sensory neurons by nanoparticles can change the activity of the central autonomic nervous system (ANS) that regulates the cardiovascular system. The evidence supporting the involvement of an autonomic neuronal-regulated pathway was also found in several ex vivo animal studies, which indicated that inhalation of nanoparticles affected cardiovascular function in response to adrenergic stimulation [5]. The earlier studies conducted by Legramante et al. and Bartoli et al. demonstrated that inhalation of either ultrafine particles from ambient air or engineered nanoparticles can alter baroreceptor reflex sensitivity, providing the evidence to support the involvement of an alteration of ANS following nanoparticle exposure [12, 13]. The information obtained from these studies all suggested an involvement of ANS in the neuronal-regulation of cardiovascular function following nanoparticle exposure.

The activity or the balance of ANS is crucial in maintaining the proper performance of the cardiovascular system. A disturbance of ANS can also lead to an increase in cardiovascular morbidity or mortality [14–16]. HRV is a proven tool for monitoring and assessing changes in ANS activity. By analysis of autonomic background of beat-to-beat interval fluctuations in the heart rate record, HRV is also an independent predictor of cardiovascular events mortality and morbidity [17, 18]. Clinical and epidemiological studies confirm a high correlation between HRV and cardiovascular disease in human studies [17, 18]. In epidemiological studies, inhalation of particular matter 2.5 μm (PM 2.5) from ambient air can trigger cardiovascular events, which were associated with a reduced HRV [19, 20]. Although most studies demonstrate that inhalation of engineered nanoparticles has adverse effects on the cardiovascular system and provided evidence for involvement of the ANS, very few studies have focused on whether exposure to nanoparticles will influence ANS outflow and the balance between the sympathetic and parasympathetic nervous system.

The objective of the present study was to determine whether and how the activity of ANS can be influenced by pulmonary exposure to MWCNTs by analysis of beat-to-beat variability of the heart rate of the electrocardiogram (EKG) recorded from freely moving rats pre-implanted with telemetry device.

## Methods

Animal. Male Sprague–Dawley [Hla :(SD) CVF] rats from Hilltop Lab Animals (Scottdale, PA, USA), weighing 275–300 g and free of viral pathogens, parasites, mycoplasmas, Helicobacter and cilia-associated respiratory (CAR) bacillus were used for all experiments. The rats were acclimated for 1 week after arrival and housed in cages ventilated with HEPA (high efficiency particulate air)-filtered air under controlled temperature and humidity conditions and a 12-h light/12-h dark cycle. Food (Teklad 7913) and tap water were provided ad libitum. The animal facilities are specific pathogen-free, environmentally controlled, and accredited by the Association for Assessment and Accreditation of Laboratory Animal Care International (AAALAC). All animal procedures used during the study have been reviewed and approved by the National Institute for Occupational Safety and Health Animal Care and Use Committee.

### Telemetry transmitter implantation

Before the surgery, rats were kept separately, quiet, and handled gently to avoid distress. Surgical instruments and supplies were autoclaved, and aseptic technique was used throughout the surgical procedure. Anesthesia was induced with 3 % isoflurane and 1 l per minute of oxygen in an induction chamber and maintained at 2 % isoflurane and ½ liter per minute of oxygen during the surgery. A temperature-controlled heating pad was used to maintain normal body temperature of the rat and was maintained via anal probe during the entire procedure. Cardiopulmonary responses were examined as an intraoperative monitoring technique along with the spinal reflexes to determine the proper depth of anesthesia. The incision sites were clipped and then aseptically prepared with povidone-iodine, followed by 70 % alcohol.

A telemetry transmitter (HD-S21, Data Sciences International, St. Paul, MN) was positioned underneath the abdominal wall on the left lateral side of the incision and was secured in place by suturing to the abdominal muscle using 4–0 non-absorbable suture (Surgical Specialties Corporation, Wyomissing, PA). Two EKG leads were tunneled subcutaneously, the negative lead secured over the right pectoral muscle and the positive lead secured at the left caudal rib region approximately 2 cm to the left of the xyphoid process. Post-operative care was strictly followed by protocol: 5 mg/kg of meloxicam (Metacam, Boehringer Ingelheim Vetmedica, Inc. St. Joseph, MO) was administered subcutaneously for pain relief, once a day for 4 days. The general condition, body weight and food and water consumption of the rats were closely monitored. Rats had a period of 3 weeks convalescence before data acquisition and inhalation exposure.

### Pulmonary MWCNTs exposure

Rats received MWCNTs by inhalation exposure. Rats were placed individually in a sealed exposure cage filled with aerosols of MWCNTs. Sham rats (control group) were placed in the chamber and exposed to filtered air. In order to minimize the stress of exposure chamber to the rat, the exposure chamber was modified from the same type of cage that is used for hosting the rats. The MWCNTs aerosol concentration was 5 mg/m^3^, and the exposure duration was 5 h. The aerosol generation system, exposure chamber and physical characterization of the MWCNTs aerosol have been described previously [21]. Previous studies have shown that this exposure scheme produced an actual pulmonary deposition of 41 μg MWCNTs in rats, which is equivalent to workers exposed to the NIOSH Recommended Exposure Level of 0.1 mg/m^3^ for about 108 workdays in a typical occupational environment [21, 22]. Although the exposure concentration used in our study was relative high, it represents the current OSHA Permissible Exposure Level. In addition, it has been observed and reported that during the production of MWCNTs, respirable particles around emission source can be higher than the concentration we used in our study [23–25].

### Data acquisition and analysis

EKG were recorded continuously for 24 h on unrestrained, conscious rats just before and during the exposure and 1 day and 7 days post-exposure, On the exposure day, rats were allowed to acclimate to the chamber for 30 min, then 5 h (9 am-2 pm) continuous EKG recordings were made during exposure. EKG data were analyzed using Ponemah software (v 5.20) and HRV time domain Marco (Data Sciences International, St. Paul, MN). HRV was evaluated by analyzing beat-to-beat variations in RR intervals. For the time domain analysis, all parameters were averaged over the course of 5 h (9 am-2 pm). For the frequency domain analysis, 5-min segments (selected from each hour over the course of 5 h) of the EKG tracing were chosen for analysis. Time series were resampled at 20 Hz and spectral power in the LF (0.25–1Hz), HF (1–3 Hz), total power and LF/HF were calculated by an HRV analysis module of Ponemah software (v 5.20).

### Statistical analysis

Data were compared using two-way (Treatment by Day) repeated measures analysis of variance. Subsequent pairwise comparisons were tested using Fishers LSD. All data were analyzed using SAS software (Version 9.3) and differences were considered statistically significant at the level of *p* < 0.05. The values in the figures were expressed as the mean ± SE.

## Results

**Fig. 1:**
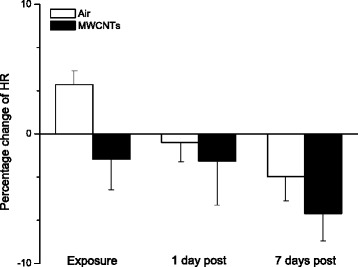
Bar graph depicting % change of HR from the basal level before exposure (Control vs MWCNTs: 328.0 ± 9.6 vs 347.0 ± 11.45 bmp). Each value represents the mean ± SE of 8 rats

Our results indicated that HR in the control group was slightly increased during exposure, whereas, the HR in MWCNTs exposure group was decreased. In Fig. 1, it compared percentage change of the HR during the exposure from pre-exposure between the control and MWCNTs-exposed groups. Although, the trend direction of the HR in response to the exposure was opposite, there was no significant difference reached between the two groups (*p* = 0.16).

**Fig. 2:**
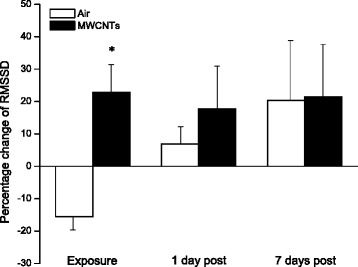
Bar graph depicting % change of RMSSD from the basal level before exposure (Control vs MWCNTs: 4.11 ± 0.39 vs 3.94 ± 0.30 ms). Each value represents the mean ± SE of 8 rats. *P* < 0.01 compared with control group (*)

**Fig. 3:**
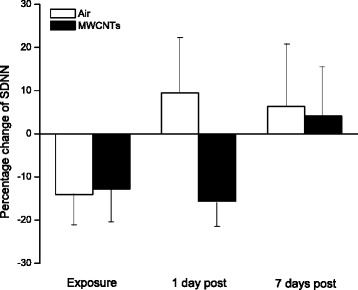
Bar graph depicting % change of SDNN from the basal level before exposure (Control vs MWCNTs: 16.79 ± 1.48 vs 18.12 ± 1.54 ms). Each value represents the mean ± SE of 8 rats

**Fig. 4:**
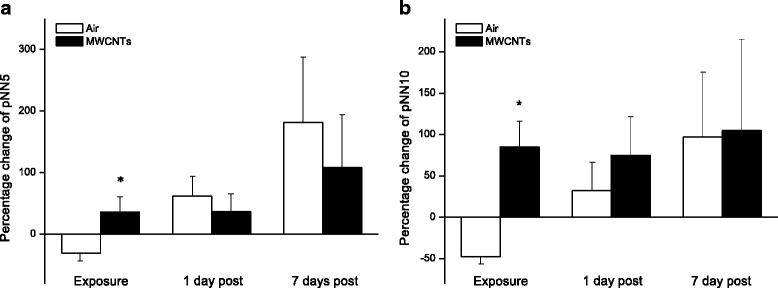
Bar graph depicting % change of pNN5 (**a**) and pNN10 (**b**) from the basal level before exposure (pNN5 (Control vs MWCNTs: 15.65 ± 3.88 vs 15.49 ± 3.18), pNN10 (Control vs MWCNTs: 3.96 ± 1.25 vs 3.31 ± 0.82)). Each value represents the mean ± SE of 8 rats. *P* < 0.01 compared with control group (*)

The indices of time domain RMMSD, SDNN, pNN5 and pNN10 were analyzed over a time course of 5 h EKG. RMMSD was increased in the MWCNTs-exposed group and slightly reduced in control group during the exposure. The percentage changes of RMMSD during exposure, 1 day and 7 days post-exposure from pre-exposure were compared between two groups. The significant difference was only observed during the exposure time period (Fig. 2). There was no difference founded between the two groups with regards to the SDNN (Fig. 3). Like RMMSD, both pNN5 and pNN10 showed significant difference between the control and the MWCNTs groups during the exposure (MWCNTs-exposed group being higher), and there was no difference observed between the two groups at 1 day and 7 days post-exposure (Fig. 4a, b).

**Fig. 5:**
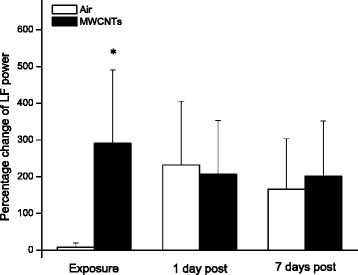
Bar graph depicting % change of LF power from the basal level before exposure (Control vs MWCNTs: 0.76 ± 0.18 vs 0.88 ± 0.22 ms^2^). Each value represents the mean ± SE of 8 rats. *P* < 0.01 compared with control group (*)

**Fig. 6:**
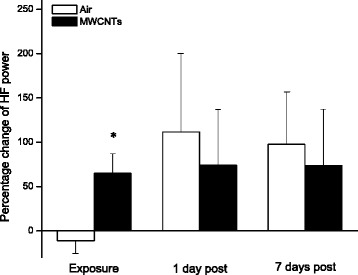
Bar graph depicting % change of HF power from the basal level before exposure (Control vs MWCNTs: 1.33 ± 0.30 vs 1.33 ± 0.28 ms^2^). Each value represents the mean ± SE of 8 rats. *P* < 0.01 compared with control group (*)

Two frequency domains, LF and HF, are the major parameters to reflect the performance of sympathetic and parasympathetic nervous system. The percentage changes of LF and HF were compared between two groups as presented in Figs. 5 and 6. Both LF and HF were significantly higher in the MWCNTs-exposed group than the control group during the exposure but there were no differences between the two groups at 1 day and 7 days post-exposure (Figs. 5 and 6).

**Fig. 7:**
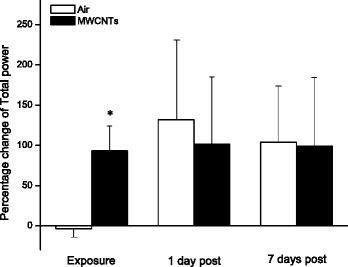
Bar graph depicting % change of total power from the basal level before exposure (Control vs MWCNTs: 2.09 ± 0.46 vs 2.21 ± 0.47 ms^2^). Each value represents the mean ± SE of 8 rats. *P* < 0.01 compared with control group (*)

**Fig. 8:**
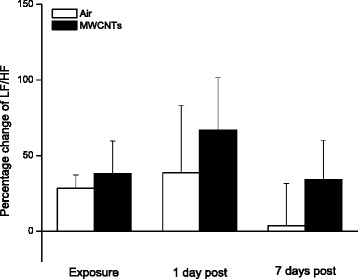
Bar graph depicting % change of LF/HF ratio from the basal level before exposure (Control vs MWCNTs: 0.59 ± 0.09 vs 0.65 ± 0.13). Each value represents the mean ± SE of 8

The total power, the sum of LF and HF power, reflects total variance in heart rate pattern over a certain length of recording and is associated with the status of the performance in autonomic nervous system. The total power was elevated during the exposure to MWCNTs (Fig. 7). The increased total power during the exposure could be influenced predominantly by HF power as shown in Fig. 6. Our study found that the LF/HF ratio was not significantly changed between the two groups during MWCNTs exposure (Fig. 8), even though the HF was dramatically increased as shown in Fig. 6.

## Discussion

The role of autonomic nervous system (ANS) in regulation of cardiovascular function has been well studied [26]. Heart rate variability (HRV), as an indicator reflecting fluctuations in the activity of sympathetic and parasympathetic nervous system, has been studied for its role in the prediction of cardiovascular morbidity and mortality in the clinical studies [17, 27]. A decrease in HRV usually indicates a worse prognosis in patients with cardiovascular event, and vise verse [27]. Recently epidemiological studies demonstrated that inhalation of PM2.5 pollutants reduces HRV and triggers cardiovascular events in a group of individuals with a pre-existing cardiovascular condition [28]. These studies suggest that exposure to particles can alter HRV, and there is an association between HRV alteration and adverse cardiovascular events. We reported previously that inhalation of engineered nanoparticles, ultrafine titanium dioxide (UFTiO_2_), alters cardiovascular function in response to adrenergic agonists [9]. However, whether inhalation of engineered nanoparticles could cause the changes in HRV has not been studied.

In the present study, we exposed the rats to engineered MWCNTs and found that inhalation of MWCNTs slightly decreased heart rate and increased the HRV indices of RMSSD, pNN5, and pNN10 (Figs. 1, 2 and 4) during the exposure, which strongly indicated that there was an alteration in autonomic nervous activity. The small increase of HR in the control group during the exposure (Fig. 1) was most likely due to a minor stress reaction when the rats were placed individually in a sealed cage. We observed that after first 30 min acclimation, the cardiovascular parameters returned to close to the level of pre-exposure, but they were still little bit higher compared to pre-exposure. For example, the blood pressure dropped rapidly from the level of entrance into the chamber after 30 min acclimation, and was about 1 % higher than pre-exposure at 1 h in control group. The blood pressure remained at this level during the rest of the exposure period. These observations suggest that a longer acclimate period may be appropriate in the future experiments. Unlike the control group, the HR was decreased slightly in the group exposed to MWCNTs. This observation could have resulted from direct stimulation of the peripheral vagus nerves by MWCNTs exposure. Increases in RMSSD, pNN5 and pNN10 could be due to activity changes in either the parasympathetic or the sympathetic nervous system. However, our study suggests that changes in RMSSD, pNN5, and pNN10 were most likely due to an increase in the activity of parasympathetic nervous system. This was supported by the significant increase in LF and HF power of the HRV in the MWCNTs-exposed group when compared with control group during the exposure period (Figs. 5 and 6). HF power is considered as an indicator of the performance of vagus nerve on the heart. Higher HF power usually indicates a domination of parasympathetic nervous activity in ANS. Other evidence to support the increased activity in parasympathetic nervous system during exposure to MWCNTs was higher LF power in the exposure group (Fig. 5). LF power usually serves as an indicator of the performance of sympathetic nerves, but in reality, LF power reflects a joint action of the vagal and sympathetic components on the heart. More importantly, the parasympathetic components can contribute to at least 50 % of LF power total components [29, 30]. Therefore, an increase in LF power could also be due to an increase in vagal nerve activity. SDNN that indicates standard deviation of normal to normal R-R intervals, was not different between control and the MWCNTs-exposed group at any time (Fig. 3). A greater SDNN usually means higher HRV; however, SDNN can remain unchanged even when the HRV is altered [31].

Interestingly, we noted that RMSSD, pNN5, and pNN10 were reduced, although not significantly, in the control group during the exposure when compared with the same group before the exposure (Figs. 2 and 4). Reduced RMSSD, pNN5, and pNN10 during the filtered air exposure could reflect astress-induced new balance between the sympathetic and parasympathetic nervous system due to reciprocal changes. The phenomenon of reciprocal changes in ANS explains why the HF power was reduced, although not significantly, in control group during the filtered air exposure (Fig. 6). This is because an increase of sympathetic nervous activity will suppress parasympathetic nervous activity, since the parasympathetic components are dominated in HF power. Therefore, the overall summation was reduced HF power as we showed in control group (Fig. 6). The stress responses including an acceleration of heart rate in control rats, as shown in Fig. 1, and an increase in systemic blood pressure was observed when the rats were placed in the exposure cages (data are not shown). In the present study, we also found that inhalation of MWCNTs resulted in an increase in total power (LF plus HF, Fig. 7) and an unchanged LF/HF ratio (Fig. 8) during the exposure. These results suggest that inhalation of MWCNTs not only changed parasympathetic nervous activity but also simultaneously altered the sympathetic nervous activity. Our study indicates that inhalation of MWCNTs and probably other engineered nanoparticles may up-regulate the activity of both sympathetic and parasympathetic nervous systems.

As shown in the Figs. 2, 3, 4, 5, 6, 7 and 8), there was no difference in HRV between the control and the MWCNTs-exposed group at 1 day and 7 days post-exposure. These findings suggest that the effect of inhalation of MWCNTs on HRV was transient, whether this transient effect can trigger a cardiovascular event consequently, and particularly in the individuals with a pre-existing cardiovascular condition, is unclear. The mechanism by which inhalation of MWCNTs induced a short alteration in HRV is most likely a quick adapted reaction occurring in either peripheral or central nervous system in response to a short-term exposure to MWCNTs. It will be interesting to determine whether this quick adaption can also occur with long-term exposure to MWCNTs.

It is generally accepted that high HRV is a sign of good adaptation, characterizing a healthy individual with efficient autonomic mechanisms; whereas low HRV is an indicator of abnormal and inadequate adaptation of the ANS, which may indicate the presence of physiological malfunction. For instance, low HRV is often reported in the individuals with mental stress or heart failure [32–34]. Stress, one of major contributing factors to cardiovascular disease, can directly increase sympathetic neuronal outflow and result in an increase of heart rate or blood pressure [35]. Heart failure, due to weak cardiac muscle and insufficient cardiac output, can increase the activity of sympathetic nervous system through peripheral baroreceptor and chemoreceptor reflexes to provide inotropic support to the failing heart and to increase the stroke volume. Increase of sympathetic nervous system activity can result in peripheral vasoconstriction which will maintain the mean arterial perfusion pressure at proper level to insure the normal function of the important organs, such as the brain. Although the mechanisms are different regarding the increase of sympathetic neuron outflow in both situations, a correlation of low HRV with high sympathetic nervous activity is common in these two different pathophysiological conditions. Although in general, high HRV is usually associated with healthy individuals, high HRV can also occur in pathophysiological conditions. It has been reported that HRV was significantly elevated in patients with congenital long QT syndrome, a heart rhythm disorder, which was associated with an abnormal over-activated parasympathetic nervous system [20, 36]. In our study, the association of a HRV alteration and cardiovascular function following MWCNT exposure is not clear. However, inhalation of engineered nanoparticles may induce alterations in ANS activity, even if transient, which may worsen already disrupted ANS in the patients with a pre-existing cardiovascular condition and trigger a cardiovascular event. This hypothesis is supported by the findings from the American Heart Association, that exposure to particulate matters <2.5 μm air pollution for only a few hours or weeks can trigger cardiovascular disease-related mortality and non-fatal events, while longer-term exposure increases the risk for cardiovascular mortality to an even greater extent than exposures over a few days [37]. Whether HRV can serve as a reliable biomarker to evaluate the performance of ANS and to predict impacts on the cardiovascular system following inhalation of engineered nanoparticles and other toxicants warrants further investigation.

## Conclusions

The observations in the present study provide fundamental evidence to support our previous findings and the hypothesis that pulmonary exposure to nanoparticles can change the outflow of ANS from the higher cardiovascular control center in medulla oblongata. In conclusion, our study indicates that inhalation of nanoparticles can induce transient alteration in the autonomic nervous system. Further studies are warranted to investigate the association between an alteration in HRV and the adverse impact of inhaled nanoparticles on cardiovascular function.

## Abbreviations

HRV: heart rate variability
MWCNTs: multi-walled carbon nanotubes
RMSSD: root mean square of successive differences
RR: inter-beat
SDNN: the standard deviation of RR interval
pNN5: the percentage of successive
RR: interval differences greater than 5 ms
pNN10: the percentage of successive RR interval differences greater than 10 ms
LF: low frequency
HF: high frequency
